# Clinical, molecular detection and phylogenetic analysis study of local foot-and-mouth disease virus in Al-Qadisiyah province of Iraq

**DOI:** 10.14202/vetworld.2018.1210-1213

**Published:** 2018-09-04

**Authors:** Khalefa Ali Mansour, Hassan Hachim Naser, Muthanna Hadi Hussain

**Affiliations:** Department of Internal and Preventive Veterinary Medicine, College of Veterinary Medicine, University of Al-Qadisiyah, Iraq

**Keywords:** cattle, clinical, foot-and-mouth disease, Iraq, polymerase chain reaction

## Abstract

**Aim:**

This study was directed during an outbreak of suspected foot-and-mouth disease (FMD) in cattle in Al-Qadisiyah province, Iraq 2016. The disease has made a huge economic loss in livestock. It was suspected that the vaccination has failed to protect the animals from the infection because of the difference in the strains. Consequently, we designed the study to make the diagnosis and detect the strain of the causative virus.

**Materials and Methods:**

The extraction of the DNA was done on 73 samples and Reverse Transcriptase Polymerase Chain Reaction (RT-PCR) was used in the detection of FMD virus (FMDV) for primary diagnosis, and serotype-specific diagnosis was done with universal primer sets 1F/1R, A-1C612, and O-ARS4 with the expected band of 329, 865, and 1301 bp, respectively.

**Results:**

Universal primer pair 1F/1R detected FMD in 55 of 73 (75.3%); of these, 37 (67.3%) were females and 18 (32.7%) were males, with high significance (p<0.01) between males and females in the PCR positivity ratio. The tested samples with positive universal primer were amplified with specific primers A-IC612 with no reaction for serotype O-ARS4.

**Conclusion:**

The products of RT-PCR were sent for RNA sequencing, and the results were 100% positive to serotype A which means that it is the predominant type in Iraq. It may help in the importing or production of the vaccine to make a preventive plan for the disease. The virus of FMD is contagious and dangerous due to its role in the huge economic loses. The detection of this virus is widely explained in lots of articles, but it is more specific and sensitive in RT-PCR and sequencing. Consequently, the authorities responsible for importing and/or production vaccines have to avoid the importing of other serotypes because it will be losing money and more outbreaks will explode.

## Introduction

Foot-and-mouth disease (FMD) is the most contagious disease of the cloven-hoofed animals and has a great potential for causing severe economic losses in susceptible farms of cattle, pigs, sheep, goats, and buffalo which are the most susceptible to the FMD. The infection of susceptible animals with FMD virus (FMDV) leads to the appearance of vesicles on the feet, in and around the oral cavity, and on the mammary gland in females [1]. The severity of the clinical signs varies with the strain of virus, the exposure dose, the age and the breed of animal, the host species, and its degree of immunity. There are seven serotypes of FMDV, namely O, A, C, SAT1, SAT2, SAT3, and Asia1, and infections with any serotype do not confer immunity against other [2].

In Iraq, the serotypes A, O, and Asia1 were recorded in the years 1952, 1957, and 1975, respectively. A severe outbreak of FMD occurred in Iraq in the period between the end of 1998 and the beginning of 1999, it affected cows, buffalos, sheep, and goats and may be present in other animals, the virus was isolated from these animals and the disease was endemic in Iraq [3]. A comprehensive epidemiological map of FMD in Iraq was recorded by Qassim and Kh [4]. Lots of outbreaks had occurred in Iraq with the last one in 2016 which made it necessary to do this research.

FMD is still prevalent in many parts of the world as emphasized by the 2001 epidemics in the European Union, southern Africa, Asia, and South America [5]. The virus of FMD has seven different serotypes: O, A, C, Asia1, SAT1, SAT2, and SAT3; within the serotypes, multiple subtypes can also be identified which sometimes fail to induce total cross-protection against other viruses of the same serotype. Genetic heterogeneity within FMDV may arise as a result of normal genetic drift [6]. This may be due to the selective pressure [7] or as a result of recombination between different FMDV genomes [8,9].

Serotype A is considered to be the most diverse of the Eurasian serotypes both genetic and antigenic [10], and 26 regional genotypes within three continental top types have been identified globally. The high level of divergence makes it difficult to prevent the disease by vaccination [11]. In this study, the owners had complained that they did vaccinate the animals, but the disease has occurred; therefore, the team of this study decided to make the identification of the serotype of the virus to find out if there is diverse between the strain of the imported vaccine and the real causative strain.

In addition, control of this disease is constantly challenged by the emergence of new virus strains. FMD is endemic in large parts of Africa, Asia, and South America. The virus can readily cross international boundaries and hence cause epidemics in previously free areas. FMD is the most economically important worldwide animal viral disease [12]. It is classified as a List A disease by “Office International des Epizooties.” The List A diseases have the potential for rapid and extensive spread within and between countries [13]. The prominent clinical signs are fever and vesicles in the mouth and feet. Although the mortality rate is very low, it is fatal in young animals. Surviving cattle subsequently carry the virus for up to 2 years. These animals will be a reservoir of the following outbreak [2].

The investigation team supposed that several herds failed to fight against the disease in spite of being vaccinated due to the diversity in strains between imported vaccine and the causative one of the virus; this study was conducted to detect the serotype of FMDV and to use the Reverse Transcriptase Polymerase Chain Reaction (RT-PCR) for the detection of the virus from samples obtained from cattle suspected to be infected in Al-Qadisiyah province, Iraq.

## Materials and Methods

### Ethical approval

The Animal Ethical Committee of the College of Veterinary Medicine, University of Al-Qadisiyah, Iraq, has approved the present study.

### Clinical examination

Being informed about the presence of an outbreak in 2016, clinical examination was done on several herds in different rural areas in the provinces: Al-Daghara, Al-Saniya, Al-Mhanawiya, Al-Shamiya, Ghammas, and Afak. The clinical signs were recorded after taking the case history of the diseases and the vaccination programs.

### Sample Collection

A total of 73 specimens were collected from the suspected cattle with infection of FMD. Vesicle fluids were packed in a frozen container and directly transported to the laboratory to be stored in the freezer until used in viral RNA extraction.

### Viral RNA extraction

The extraction of viral RNA from the specimens was done using AccuZol™ RNA Extraction Kit Bioneer, Korea. A 250 µl of the sample was placed in 1.5-ml microcentrifuge tube, and then, 1-ml trizol reagent was added and mixed well by vortex for 1 min. After that, 200 µl chloroform being added and mixed powerfully for 15 s, and then, the mixture incubated on ice for 5 min. The tubes placed in a cold centrifuge (4°C) at 12,000 rpm for 15 min. The supernatant was transferred to a new microcentrifuge tube, 500 µl isopropanol was added and the combination was mixed by inverting the tube 4-5 times and incubated at 4°C for 10 min. The tubes were returned to centrifuge at 12,000 rpm for 10 min, and then, the supernatant was discarded. The RNA pellet was washed by adding 1 ml of 80% ethanol with diethyl pyrocarbonate (DEPC) and mixed again and placed in centrifuge at 12,000 rpm for 5 min. After that, the supernatant was discarded and the RNA pellet left to air dry. Finally, 50 µl DEPC water was added to the elution of RNA pellet, and then, the extracted RNA sample was checked by Nanodrop spectrophotometer and stored in −20°C freezer until used in RT-PCR assay.

Reverse-transcription PCR has performed for detecting and genotyping of FMDV using the one-step RT-PCR kit (Accu Power^®^ RT-PCR PreMix from Bioneer, Korea). This method was defined by Sehrish *et al*. [14]. The primers were designed and provided by Bioneer Company, Korea, as shown in Table-1. The RT-PCR master mix was prepared according to the instructions of the company as illustrated in Table-2.

**Table-1:** The primers of the cDNA of the FMDV.

Primer	Sequence		Amplicon
Universal FMD primer	F R	GCCTGGTCTTTCCAGGTCT CCAGTCCCCTTCTCAGATC	330 bp
O genotype primer	F R	TBGCRGGNCTYGCCCAGTACTA CCAGTCCCCTTCTCAGATC	1150 bp
A genotype primer	F R	TACCAAATTACACACGGGAA CCAGTCCCCTTCTCAGATC	865 bp

FMDV=Foot-and-mouth disease virus

**Table-2:** The components of the master mix reaction of RT-PCR.

RT-qPCR master mix	Volume
Template of RNA	5 mL
Forward primer (10 pmol)	2 mL
Reverse primer (10 pmol)	2 mL
DEPC water	11 mL
Total	20 mL

DEPC=Diethyl pyrocarbonate, RT-PCR=Reverse-transcriptase polymerase chain reaction

These RT-PCR master mix reaction components that mentioned in Table-2 were added into standard RT-PCR premix tube containing RocketScript Reverse Transcriptase Enzyme, DNA polymerase, dNTPs, and 10× buffer as well as loading dye. Then, all RT-PCR master mix tubes placed in ExiSpin vortex centrifuge for 3000 rpm for 3 min, after that transferred into Mygene PCR thermocycler. RT-PCR thermocycler conditions reverse-transcription step, and PCR amplification was done in one tube reaction by applying the following RT-PCR thermocycler conditions in Table-3.

**Table-3:** Thermocycler conditions of the RT-PCR.

Step	Condition	Cycle
Reverse transcriptase	50°C for 15 min	1
Initial denaturation	95°C for 5 min	1
Denaturation	95°C for 30 s	35
Annealing	56°C for 30 s	
Extension	72°C for 1 min	
Final extension	72°C for 10 min	1
Hold	4°C	Finish

RT-PCR=Reverse-transcriptase polymerase chain reaction

RT-PCR products were examined through the agarose gel electrophoresis; 2% agarose was prepared using 1× Tris-Borate-EDTA buffer, and it was stained with ethidium bromide at 100 V and 80 am for 1 h. The RT-PCR bands were seen under U.V transilluminator.

## Results

### Clinical examination

The clinical examination revealed that most of the suspected cases showed the same clinical signs such as reduced milk production with high fever (40-41°C), accompanied by anorexia, and followed by acute painful stomatitis. At this stage, the temperature reaction has raised. There was excessive salivation, saliva drooling, ropy strings, a specific smacking of the lips, and the animal chews painfully. Lameness with the presence of painful lesions on the feet had made the animals annoyed. Fluid-filled vesicles and bullae (about 1-3-cm diameter) appeared in the buccal cavity, dental pad, and tongue. These always ruptured within 24 h, leaving an awful raw surface.

### RT-PCR and serotype detection

**Figure-1:**
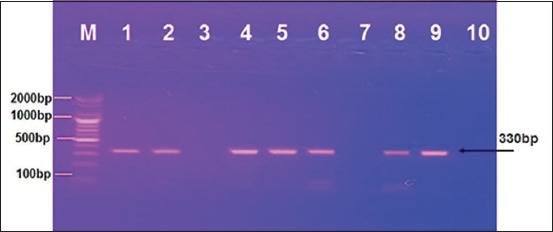
Agarose gel electrophoresis; positive bands of universal foot-and-mouth disease virus in samples 1, 2, 4, 5, 6, 8, and 9.

**Figure-2:**
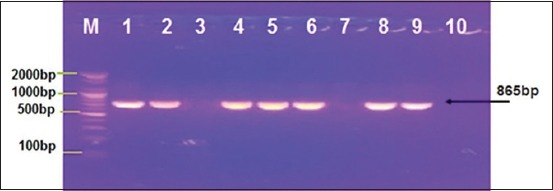
Agarose gel electrophoresis; positive bands of serotype A virus of foot-and-mouth disease in samples 1, 2, 4, 5, 6, 8, and 9.

A total of 73 samples were tested in RT-PCR for initial screening, and 55 samples were detected positive in RT-PCR as universal FMDV as shown in Figure-1. The same samples were examined for specific primers of serotype A and O; all were positive to the type A as shown in Figure-2.

### DNA sequencing and phylogeny

Direct sequencing was performed on RT-PCR products. According to Sanger dideoxy sequencing method for the confirmative diagnosis of serotype in the outbreak [15], the RT-PCR product was sent to the National Center for Biotechnology Information (NCBI) GenBank for sequencing, and the result confirmed the positivity of the presence of the virus by finding the polyprotein gene, partial cdc at 329 bp. The result was received by an E-mail containing the accession number KY662045 on May 26, 2017.

**Figure-3:**
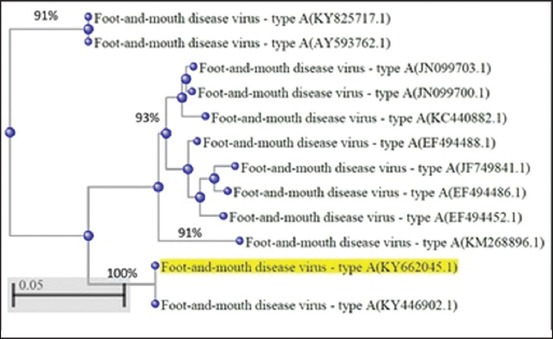
Phylogenetic tree analysis of foot-and-mouth disease virus type A with several closed strains characterized previously in neighbor countries.

BLAST analysis of these directly sequenced RT-PCR products discovered that (A) serotype of FMD was present in all positive samples from the outbreaks ensuring the FMDV as the causative agent for these outbreaks as shown in Figure-3 which reveal the tree analysis of the virus with others recorded in other countries.

## Discussion

FMD is one of the most economically demoralizing diseases of ruminants all over the world [16]. In this study, the disease has resembled the outbreaks in other countries including the spread and the clinical signs; the results of the clinical examination had come true with other published articles like that in David *et al*. [17]. The illegal trade of animals across the borders of countries surrounding Iraq has remained a major problem for disease management. Several tests are routinely used for the early diagnosis and subsequent actions for the control and eradication of infectious diseases.

The universal primer pairs IF/IR and P1/P2-based RT-PCR along with sequence detection can be used in the primarily rapid detection of FMD in infected animals [18]. It was recorded to be very successful for the detection and confirmation of FMDV serotype directly from the samples. Consequently, FMD A serotype was found to be heritably similar to neighboring countries such as Turkey, Pakistani, and Egypt as well as other serotypes detected previously in other provinces in Iraq.

Besides, network analysis of the isolate RNA in the website of the NCBI revealed great relation between the detected virus and others; it was completely related to serotype A in Pakistan, 2017 (KY446902), and the homology was 93% with viruses detected in Basrah, Iraq, 2016 (JN099703), and Pakistan 2016, (EF494452). At the same time, detected virus was allied 92% with viruses diagnosed in Pakistan 2006 (EF494488), Egypt 2012 (KC440882), Iraq 2017 (KY825717), and Turkey 2006 (JF749841), while it was similar to viruses isolated in Iraq 2005 (AY593762) and Turkey 2013 (KM268896) with 91%.

The reverse transcription RT-PCR is highly sensitive and specific test ever used in the detection of FMDV. Therefore, it was used in this study as well as previous studies in which outbreaks were detected in Iraq like that in Basrah [19]. The outbreak may happen due to virus escape from vaccination, or incomplete inactivation or other causes related to vaccine quality, and may also be happened due to the difference between the serotype of the imported vaccine and the present serotype in the region where the outbreak had happened. Consequently, the administrative policies should take this information in regard to the importation and/or production of the vaccines. Finally, it was highly significant (p<0.05) between males and females infected in the outbreak, and it may be related to the presence of lots of females in the herds 90-95% due to the economic benefit more than the males.

## Conclusion

The results were 100% positive to serotype A which means that it is the predominant infective type in Iraq. It may help in importing or production of the vaccine to make a preventive plan for the disease. Consequently, the authorities responsible for importing and/or production vaccines should avoid the importing of other serotypes because they shall be losing money and more outbreaks may explode.

## Authors’ Contributions

KHA and MHH have supposed the problem and designed the work. They did the survey and collected the samples. HHN carried out the laboratory tests. All authors collected references about the subject. All authors read and approved the final manuscript.
